# A bifurcation of the peak: new patterns of traffic peaking during the COVID-19 era

**DOI:** 10.1007/s11116-022-10329-1

**Published:** 2022-09-09

**Authors:** Yang Gao, David Levinson

**Affiliations:** School of Civil Engineering, The University of Sydney, Sydney, New South Wales Australia

**Keywords:** COVID-19, Traffic flow, Diurnal curve, Morning commuting, Double-humped phenomenon, Commuting composition

## Abstract

This paper analyzes the emergence of two well-defined peaks during the morning peak period in the traffic flow diurnal curve. It selects six California cities as research targets, and uses California employment and household travel survey data to explain how and why this phenomenon has risen during the pandemic. The final result explains that the double-humped phenomenon results from the change in the composition of commuters during the morning peak period after the outbreak.

## Introduction

Morning commuting is a critical problem in traffic congestion theory, and since (Vickrey 1969), the morning commuting has been extensively studied. The morning peak period is important because most recurrent delay and incident delay caused by congestion occurs during this period (Noland and Small 1995), and commute trips are the main component of morning period travel (Levinson and Krizek 2007). In order to avoid the more expensive cost of being late due to congestion, commuters could choose to travel earlier in the morning to avoid the peak (Parthasarathi et al. 2011). The impact of the COVID-19 outbreak on morning peak traffic flow changed these calculations. In the face of COVID-19, many governments adopted the strategy to encourage or require people to stay at home as much as possible and obtain daily necessities through delivery services, family or friends (Daniel 2020). The self-isolation or lockdown imposed by the authorities reduced work or education travel (Abdullah et al. 2020), and caused a decline in traffic flow.

As the pandemic worsened, authorities began to enforce lockdown orders and travel restrictions. Throughout the United States, many cities and states issued travel restrictions, requiring residents to limit their travel to food, medicine, medical care, and work deemed ‘essential’, and as commuting and social travel decrease, driving speed on the road increased, and travel time decreased accordingly (Parr et al. 2020). In areas where there is a stay-at-home order in the United States, the average daily travel distance in late March 2020 dropped from 8.0 to 1.6 km, which has undoubtedly decreased the traffic flow and reduced traffic congestion (Glanz et al. 2020). Similar lockdown orders and travel restrictions are applied by governments all over the world, such as Canada (Tian et al. 2021), Britain (Robinson et al. 2021), France (Di Domenico et al. 2020), China (Lau et al. 2020), India (Lancet 2020), Australia (Chow et al. 2020), etc.

Regardless of subjective or objective reasons, the number of travelers drastically reduced after the outbreak, which changed traffic flow, especially during the morning peak travel period, as reflected in the traffic flow diurnal curve during the morning peak period. In the traditional traffic flow diurnal curve, the triangular shape occurs during the morning and evening peaks. The morning peak is more pronounced, because the main component of travel during this period is commuting to work and school (Parthasarathi et al. 2011).

And due to the impact of the pandemic, the number of commuters in the morning peak period undoubtedly dropped both due to unemployment or work from home. Overall, 25% of United States adults reported that someone in their family was fired or unemployed due to the COVID-19 outbreak, and 15% of them said it happened to them personally (Parker et al. 2020). Moreover, 37% of jobs in the United States can be performed at home, and there are significant differences between cities and industries (Dingel and Neiman 2020). These data indicate that due to the outbreak of the pandemic, there should have been a large number of commuters lost during the morning peak, and this was indeed the case. In addition to commuting loss, the changes in the composition of morning peak commuting emerge, because different industries have different shares of workers that can work from home (denoted as WFHc for short) (Dingel and Neiman 2020; Cetrulo et al. 2020; Bloom et al. 2015), and capital-intensive industries such as information, management, education, finance, insurance, science and technical services account for a higher share of WFHc jobs than labor-intensive industries (Dingel and Neiman 2020). We expect this further changes the triangular shape that existed in the traffic flow diurnal curve pre-COVID.

The traffic flow peak phenomenon during the morning peak period has significant impacts on the choice of the departure time of travelers (He 2013), the determination of the travel cost function (Tian et al. 2010), and the formulation of macroscopic transportation policies (Calthrop et al. 2000), such as parking, road pricing, congestion pricing etc. If the traffic pattern of this triangular shape changes due to the COVID-19, the applications mentioned above will also be affected, so it is necessary to explore whether the traffic pattern during the morning peak period changes significantly before and after the COVID-19. A previous study (Gao and Levinson 2021) found that the traffic flow diurnal curve of the Minneapolis-St. Paul freeway network in Minnesota changed from the previous single peak (triangular shape) to the double peak after the outbreak of the COVID-19. However, to date there has been no detailed analysis on whether this Double-Humped phenomenon is general in other regions, and its quantification and specific causes. Therefore, in this paper, we continue to investigate this Double-Humped phenomenon in other cities, taking California as our data set because of its high quality traffic performance measurement system (PeMS) data availability, and because it is a large state with multiple metropolitan areas, and give a specific quantitative method and cause analysis.

The remainder of this paper is organized as follows: section “Methodology” elaborates on the quantification methodological framework of the Double-Humped phenomenon. Section “Field data analysis” collects field traffic flow and speed data in six cities selected in California to obtain the diurnal curves of the traffic flow and unit travel time before and after the outbreak. Section “Causal analysis” explains the changes in the Double-Humped phenomenon through the collection and analysis of monthly average employment data and California Household Travel Survey (CHTS) data. Section “Conclusions” concludes.

## Methodology

The methodology of this paper mainly focuses on the acquisition of traffic flow and unit travel time diurnal curves for city’s freeway network, shown in section “Traffic flow and unit travel time diurnal curves acquisition”, and the peak quantification in traffic flow diurnal curves, shown in section “Peak quantification in the diurnal curve”, including peak candidate set determination (section “Peak candidate set determination”) and Double-Humped quantification (section “Double-Humped quantification”).

### Traffic flow and unit travel time diurnal curves acquisition

The traffic data of a single day may be affected by accidents or loop detector faults, which may cause large deviations. In order to avoid the noise inherent in a single-day’s data, the network traffic flow adopts the daily average method to obtain the daily trend of the traffic time series for a continuous period of time (Li et al. 2015). The diurnal curve, recorded with traffic aggregated to 5-min periods, is averaged over multiple days and stations. The average of almost evenly distributed detector stations on the freeway network provides an indicator of the overall traffic flow level of the city’s freeway network, and the formula is shown in Eq. (1) below.

1 $$\begin{aligned} Q(t)=\frac{1}{M}\cdot \frac{1}{N}\sum _{m=1}^{M}\sum _{n=1}^{N}q_{mnt} \end{aligned}$$

where *t* is 5-min departure time interval, *M* is the number of days, *N* is the number of stations in the selected network and $q_{mnt}$ is the traffic flow measured by the loop detector station *n* at departure time *t* on day *m*.

The network unit travel time (Yildirimoglu et al. 2015) is the travel time (min) spent per kilometer in the selected network, which represents the extent of traffic congestion. The daily average method is also used to eliminate data errors, as shown in Eq. (2) below.

2 $$\begin{aligned} T(t)=\frac{1}{M}\cdot \frac{1}{N}\sum _{m=1}^{M}\sum _{n=1}^{N}\frac{1}{v_{mnt}} \end{aligned}$$

where 5*t* is 5-min departure time interval, *M* is the number of days, *N* is the number of stations in the selected network and $v_{mnt}$ is the traffic speed measured by the loop detector station *n* at departure time *t* on day *m*.

### Peak quantification in the diurnal curve

For the obtained diurnal curves of network traffic flow, we need to quantify the peak in these curves to better understand the traffic patterns in the urban network. Some related research has been completed in recent years (Xiao et al. 2018, 2011; Palshikar et al. 2009; Du et al. 2006), and the methods mainly focus on geometric analysis and machine learning algorithms to complete the peak quantification in huge data sets. Due to the relatively small scale of the data set in the urban traffic diurnal curve, the peak quantification method will mainly focus on geometric analysis in this paper, and the first step is to determine the peak candidate set ${\mathcal {C}}$.

#### Peak candidate set determination

If a point $p_t$ in diurnal curves has the potential to be a peak, the first condition it needs to meet is that the slope on the left is positive and the slope on the right is negative, as described below.

3 $$\begin{aligned} S(p_t)= \left\{ \begin{array}{l} S(p_t)_l=\frac{y_{p_t}-y_{p_{t-1}}}{x_{p_t}-x_{p_{t-1}}}>0\\ S(p_t)_r=\frac{y_{p_{t+1}}-y_{p_t}}{x_{p_{t+1}}-x_{p_t}}<0\\ \end{array} \right. \end{aligned}$$

where $S(p_t)_l$ is the left slope of the point $p_t$, $S(p_t)_r$ is the right slope of the point $p_t$, $y_{p_t}$ is the y-axis coordinate value of the point $p_t$, and $x_{p_t}$ is the x-axis coordinate value of the point $p_t$.

According to Eq. (3), we can determine that when $S(p_t)_l$ is greater than 0, and $S(p_t)_r$ is less than 0, the point $p_t$ is a peak candidate point, and further determine the peak candidate set ${\mathcal {C}}$ shown below.

4 $$\begin{aligned} {\mathcal {C}}=\{p_{1},p_{2}\ldots p_{n}\} \end{aligned}$$

#### Double-Humped quantification

**Fig. 1:**
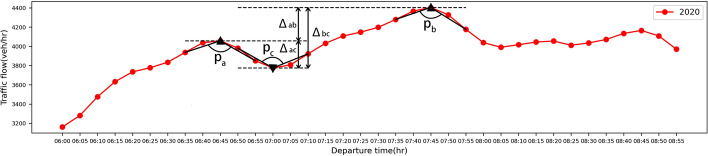
Traffic flow diurnal curve of Los Angeles in 2020

After obtaining the peak candidate set ${\mathcal {C}}$, we need to further perform Double-Humped quantification. Here, the diurnal curve of traffic flow in Los Angeles is taken as an example as shown in Fig. 1.

According to the diurnal curves of traffic flow in all cities, we observe that the traffic flow has two peaks at around 6:45 and 7:45 am, and a valley, which has a negative left slope and positive right slope, at around 7:00 am, such as shown in the traffic flow diurnal curve of Los Angeles in 2020 Fig. 1. This Double-Humped phenomenon has become more significant after the outbreak of the pandemic, and we will quantify this Double-Humped phenomenon to demonstrate it.

We divide the peak candidate set ${\mathcal {C}}$ into two subsets: ${\mathcal {C}}_1$ and ${\mathcal {C}}_2$, the corresponding peak candidate points before and after 7:00 am. Then, we select the points $p_{a}$ and $p_{b}$ with the largest traffic flow from these two subsets as two peak points in the diurnal curve, and choose the valley point $p_{c}$ between the two peaks $p_{a}$ and $p_{b}$, shown in Fig. 1. Next, we will use these three points to quantify the Double-Humped phenomenon as follows.

**Fig. 2:**
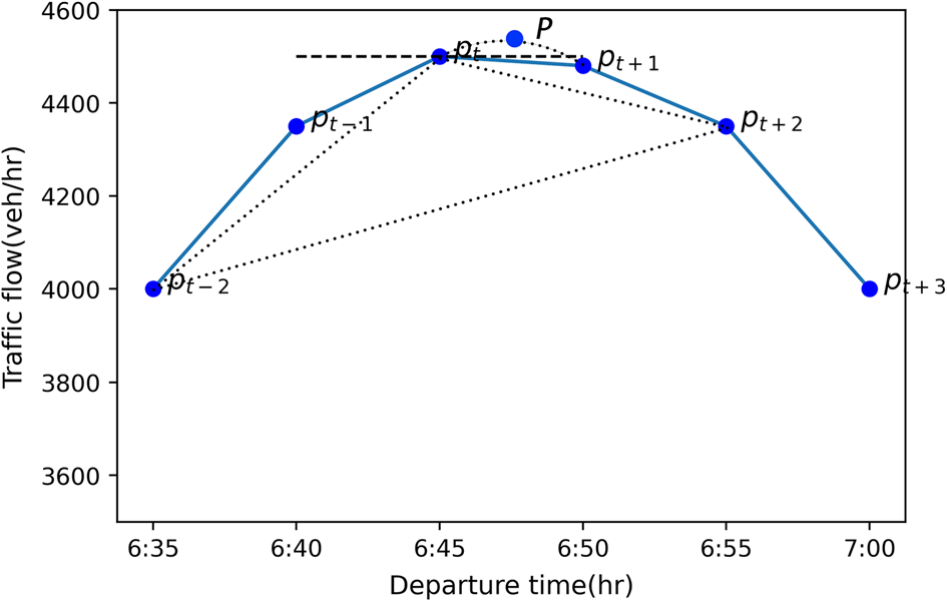
Diurnal curve example

First, we will calculate the acuteness (*A*) of three points $p_{a}$, $p_{b}$, and $p_{c}$ separately. Acuteness is defined by the cosine of the angles formed by the points and the diurnal curve parts on both sides shown in Fig. 1. Considering that the time interval of the data in this paper is 5 min, the true peak point may exist in the time interval, as shown in Fig. 2 below.

In Fig. 2, if the acuteness of the candidate point $p_t$ is calculated according to the angle formed by the line segments $p_{t-1}p_t$ and $p_tp_{t+1}$, the result will have a large deviation with the true value of the peak point *p* existing in the time interval. Therefore, the acuteness calculation needs to be expanded to a suitable range. In the case of large-scale and small-interval data (Xiao et al. 2018), this range has been shown as line segments formed by between 3 and 10 points on the left and right sides of the peak point to obtain good test results. Considering the small-scale and large-interval of the diurnal curves data set in this paper, the calculation range of the acuteness (*A*) of point $p_t$ is extended to the line segment formed by the second adjacent points, which are $p_{t-2}p_t$ and $p_tp_{t+2}$, as shown in Eq. (5) below, which is essentially the cosine law and is used to calculate the cosine, i.e. acuteness, of the angle formed by the line segments.

5 $$\begin{aligned} A(p_t)=\frac{(D_{p_tp_{t-2}})^2+(D_{p_tp_{t+2}})^2-(D_{p_{t-2}p_{t+2}})^2}{2\cdot D_{p_tp_{t-2}}\cdot D_{p_tp_{t+2}}}, \in [-1,1] \end{aligned}$$

where the y-axis length unit is based on the flow data, and the x-axis length unit is $10 \cdot {\textit{time}}$ in minutes, because the difference in the magnitude of the flow is on the order of $10^2$, so this can better equalise nominal units to reflect the change in acuteness. $D_{p_tp_{t-2}}$ is the ‘length’ of the line segment $p_tp_{t-2}$, $D_{p_tp_{t+2}}$ is the ‘length’ of the line segment $p_tp_{t+2}$ and $D_{p_{t-2}p_{t+2}}$ is the ‘length’ of the line segment $p_{t-2}p_{t+2}$. The ‘length’ (*D*) is in units of decaminutes in the x-axis and veh/hr in the y-axis

According to Eq. (5), we can get the acuteness of the points $p_{a}$, $p_{b}$, and $p_{c}$ in diurnal curves. In addition to having greater acuteness at the peak and valley points, a significant Double-Humped curve also has requirements for the peak value. The more prominent and closer the double peaks the more significant the Double-Humped curve, as shown in Fig. 1, which is reflected in the $\Delta _{ac}$ and $\Delta _{bc}$ as large as possible, and $\Delta _{ab}$ as small as possible. This is quantified with the Double-Humped phenomenon index *I* shown below.

6 $$\begin{aligned} I=\frac{2+A(p_{a})+A(p_{b})}{1-A(p_{c})}\cdot \frac{\Delta _{ac}+\Delta _{bc}}{\Delta _{ab}}, \in [0,+\infty ] \end{aligned}$$

where $\Delta _{ab}$ is the traffic flow difference between the double peaks, $\Delta _{ac}$ is the traffic flow difference between the first peak and the valley, $\Delta _{bc}$ is the traffic flow difference between the second peak and the valley, and the larger the *I*, the more obvious the Double-Humped phenomenon of the diurnal curve.

## Field data analysis

**Fig. 3:**
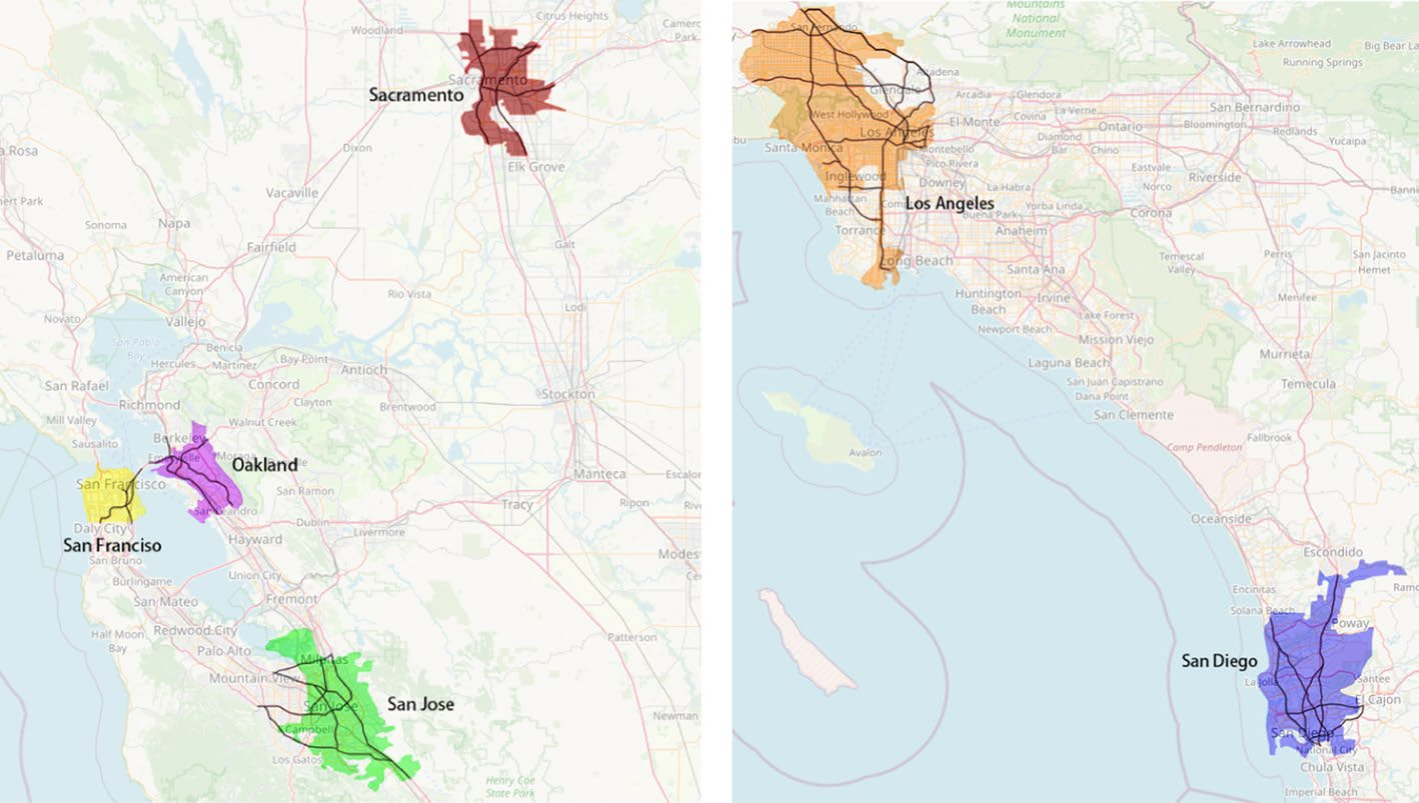
The freeway networks of selected six cities in California

We chose as research sites the freeway networks of six cities in California: San Diego, San Francisco, Sacramento, Los Angeles, Oakland and San Jose. Because this paper aims to explore changes in traffic patterns during the morning peak period in selected cities before and after the pandemic, we choose four years of data before and after the COVID-19 outbreak as the data source. The selected morning peak period runs from 6:00 to 9:00 am and the 5-min loop detector data for all working days from March 1 to July 31 in 2018, 2019, 2020 and 2021 come from the state’s freeway performance measurement system (PeMS) (California Department of Transportation 2021). The freeway networks of the selected cities are shown in Fig. 3 below.

**Fig. 4:**
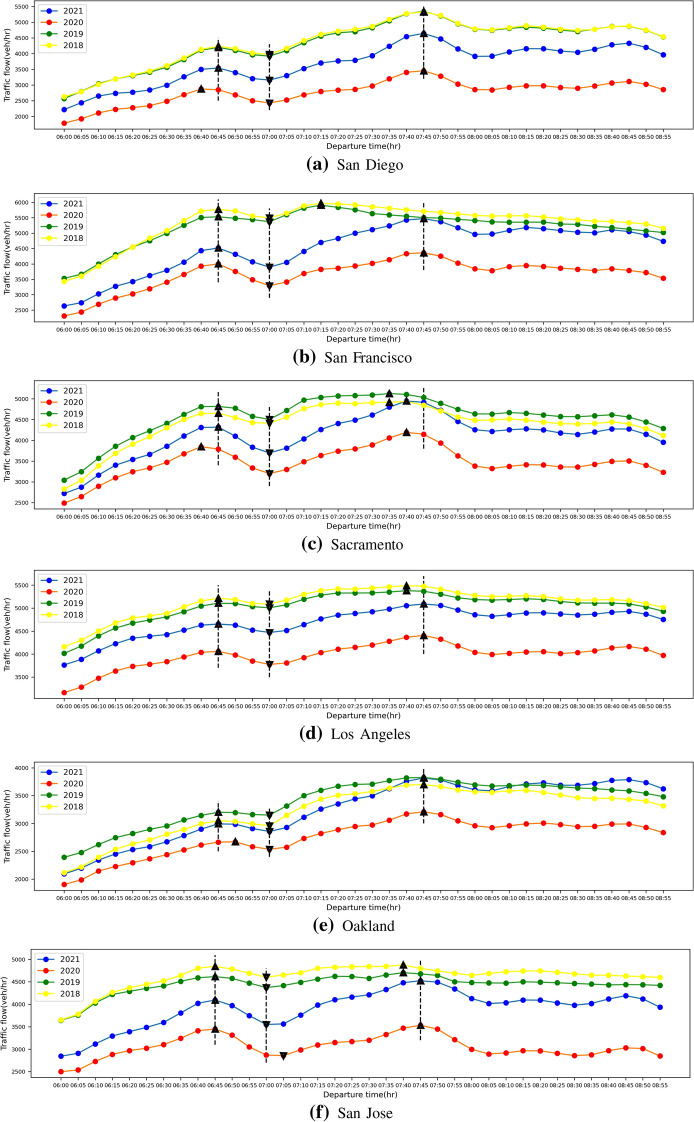
The traffic flow diurnal curves during the morning peak period for six cites in 2018, 2019, 2020 and 2021

**Fig. 5:**
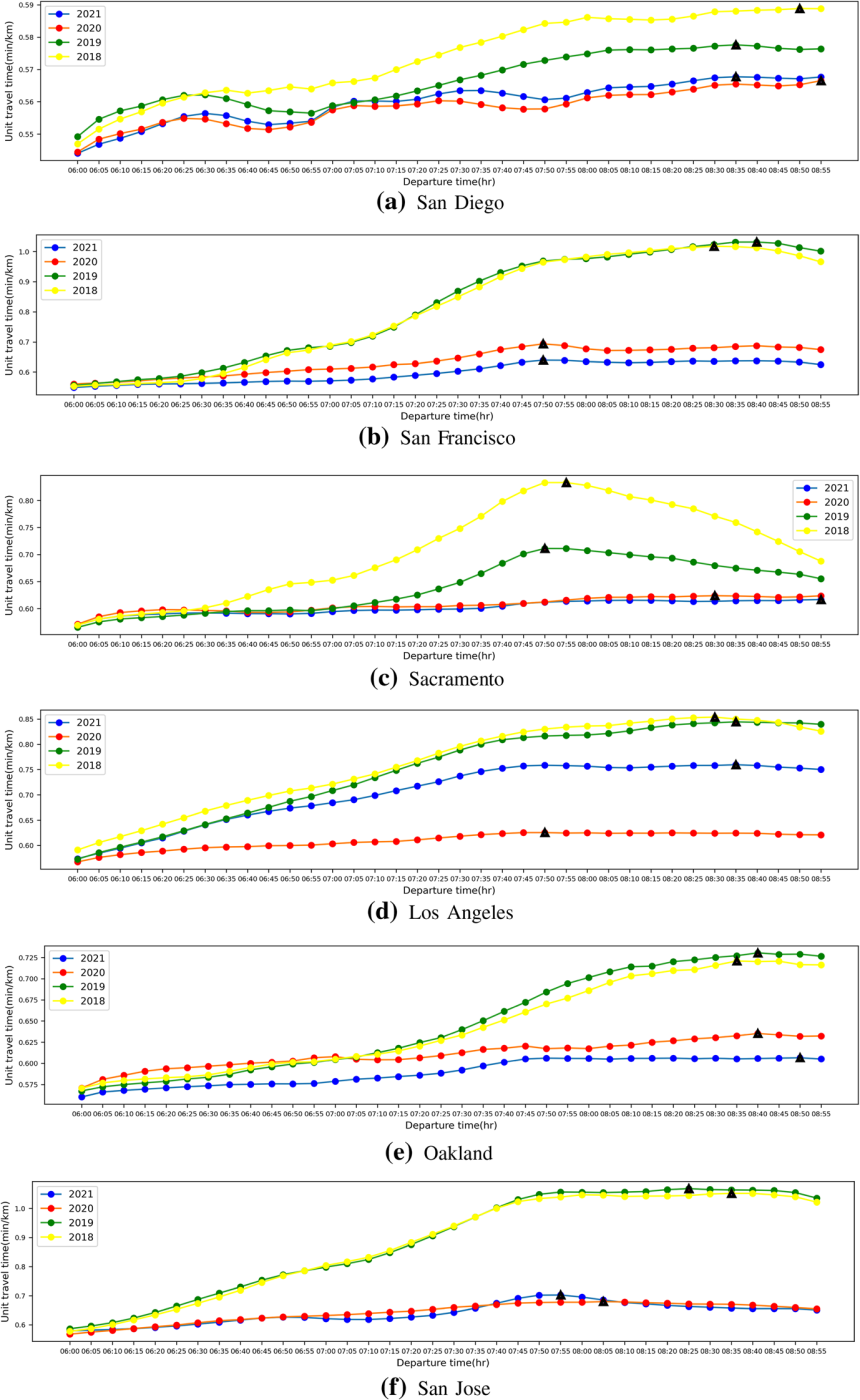
The unit travel time diurnal curves during the morning peak period for six cites in 2018, 2019, 2020 and 2021

The traffic flow diurnal curves and unit travel time diurnal curves during the morning peak period for these six cites in 2018, 2019, 2020 and 2021 are shown in Figs. 4 and 5 below.

From Figs. 4 and 5, we observe that there is an obvious Double-Humped phenomenon in the diurnal curves of traffic flow, but it is not obvious in the unit travel time diurnal curves, because the travel time is related to the speed of travel, and the change of speed is not only related to the traffic flow, but also to the density of vehicles already on the road (i.e. entering the road in previous time periods). Therefore, we only compare the peak values of the unit travel time diurnal curves without further calculating Double-Humped phenomenon index *I*. The comparison of these results is shown in the table below.

**Table 1:**
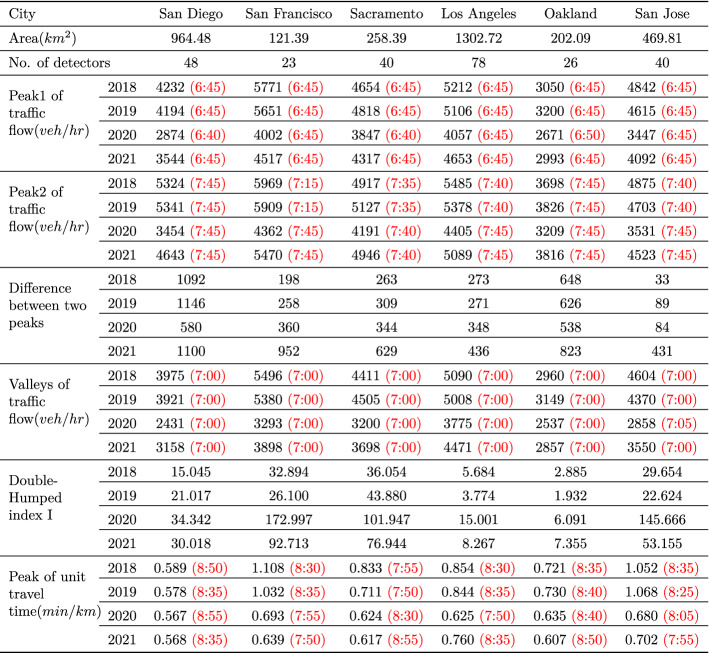
Comparison of the results of the freeway network average traffic flow and unit travel time diurnal curves in six cities in 2018, 2019, 2020 and 2021

Black indicates value, Red indicates time of day when value occurs

**Fig. 6:**
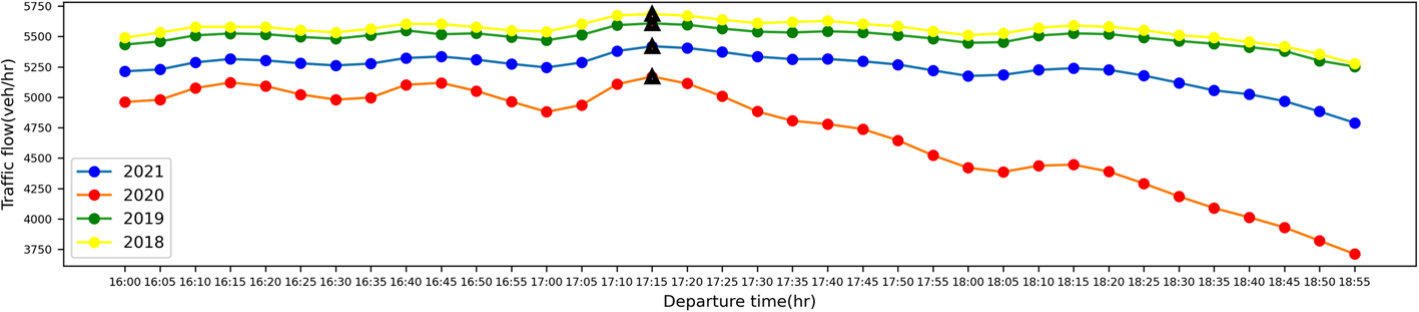
The traffic flow diurnal curve during the evening peak period for Los Angeles in 2018, 2019, 2020 and 2021

From Table 1, we observe that in 2018 and 2019 before the COVID-19 outbreak, traffic patterns during the morning peak period were largely consistent, including the magnitude and time of occurrence of traffic flow peaks, Double-Humped index I, and the magnitude of peak of unit travel time and its time of occurrence. In addition, we also notice that during the morning peak period, in the six cities selected, the traffic flow double-peaks occurred at almost the same time, except for San Francisco, whose second peak occurred about 30 min earlier than the other cities. We posit the reason is that, according to employment data provided by the California Employment Development Department (EDD), the proportion of San Francisco’s labor force engaged in information technology is around 30%, while this value is only about 10–20% in other cities, and this different workforce composition makes the occurrence time of its second peak different from other cities. After the outbreak of the pandemic, since most of the information technology industry employees can choose to work remotely from home (Dingel and Neiman 2020), it has caused a large loss of the number of commuters during the morning peak period, and the occurrence time of the second peak has been delayed by 30 min in San Francisco. Compared with before the outbreak of COVID-19, the peak average traffic flow of the urban freeway network decreased in 2020 and began to recover in 2021, but it was still lower than the value before the outbreak, which also led to a lower peak of unit travel time than before the outbreak. This shows that due to the pandemic, the congestion during the peak period has been alleviated. In addition, the Double-Humped phenomenon index *I* of 2020 and 2021 has increased apparently compared with 2018 and 2019, which indicates that the Double-Humped phenomenon has become very significant because of the pandemic. We are also interested in whether there is a double-humped phenomenon in the evening peak period, and therefore, combined with the data from Los Angeles, the traffic flow diurnal curves during the evening peak period before and after the outbreak of COVID-19 are shown in Fig. 6 below.

From Fig. 6, we observe that traffic flow during the evening peak period also decreased significantly in 2020 after the COVID-19 outbreak, and also recovered in 2021, which is consistent with the change in traffic flow during the morning peak period, but the traffic flow during the evening peak period recovered faster in 2021. We posit this is because of the lower share of commuting (with its continued work-from-home substitution) in the evening and the higher share of other activities, such as shopping, exercising, etc. (Karlamangla 2021). In addition to this, we can also find there is a much weaker Double-Humped phenomenon during the evening peak period, which is due to the fact that commuting purposed trips account for a lower proportion of travel during the evening peak period compared to the morning peak period, and the travel distribution is more spread out, making it difficult to form significant peaks (Parthasarathi et al. 2011; Winick et al. 2008).

## Causal analysis

For the significant Double-Humped phenomenon in the diurnal curve of traffic flow in the post-COVID-19 era, we posit the change in the composition of the commuting workforce is the root cause (Gao and Levinson 2021). From Table 1, we find after the outbreak of the pandemic, the traffic flow during the morning peak period has dropped severely, and the main component of travel during this period is commuting. Therefore, the reduced traffic flow can be divided into two parts, which are people who lost their jobs because of the pandemic and people who did not lose their jobs but worked from home due to personal reasons or restrictions.

Due to the outbreak of COVID-19, each industry category has a part of workers who can work from home (WFH), and the corresponding capacity (referred to as WFHc) also has a weight (Dingel and Neiman 2020; Cetrulo et al. 2020; Bloom et al. 2015). For example, capital-intensive industries such as information, science, education, finance, business management, and technical services have higher weights, while labor-intensive industrie such as agriculture, mining, construction, fishing and hunting, accommodation, retail trade, and food industry have lower weights (Dingel and Neiman 2020). Therefore, we collected monthly average employment data for various industries from the EDD in 2018, 2019, 2020, and 2021, and the corresponding WFHc weight from Dingel and Neiman (2020), as shown in Table 3.

According to the table, the monthly average employment of most industries has been reduced by varying degrees in different cities after the outbreak, and this impact has ameliorated in 2021. This situation is the same as the change of traffic flow peaks after the outbreak in Table 1. Therefore, we can use the data in Table 3 to calculate the commuters loss extent index *L* for each city due to the COVID-19 outbreak, as shown in Eq. (7) below.

7 $$\begin{aligned} L=\frac{\sum _{k=1}^{K}0.5\cdot W_{k}\cdot ( E_{k}^{2020}+E_{k}^{2021})+\sum _{k=1}^{K}(0.5\cdot (E_{k}^{2018}+E_{k}^{2019})-0.5\cdot (E_{k}^{2020}+E_{k}^{2021}))}{\sum _{k=1}^{K}0.5\cdot (E_{k}^{2018}+E_{k}^{2019})} \end{aligned}$$

where *K* is the number of the industries in Table 3, $W_k$ means the WFHc weight of the *k*th industry in Table 3, $E_{k}^{2018}$, $E_{k}^{2019}$, $E_{k}^{2020}$, and $E_{k}^{2021}$ mean the monthly average employment of the *k*th industry in 2018, 2019, 2020 and 2021 respectively.

We also calculate the change rate $\Delta I$ of the Double-Humped phenomenon index *I* for different cities after the outbreak, as shown below.

8 $$\begin{aligned} \Delta I=\frac{0.5\times (I_{2020}+I_{2021})-0.5\times (I_{2018}+I_{2019})}{0.5\times (I_{2018}+I_{2019})} \end{aligned}$$

where $I_{2018}$, $I_{2019}$, $I_{2020}$, and $I_{2021}$ mean the Double-Humped phenomenon index *I* of traffic flow diurnal curves for each city in 2018, 2019, 2020, and 2021 respectively.

**Fig. 7:**
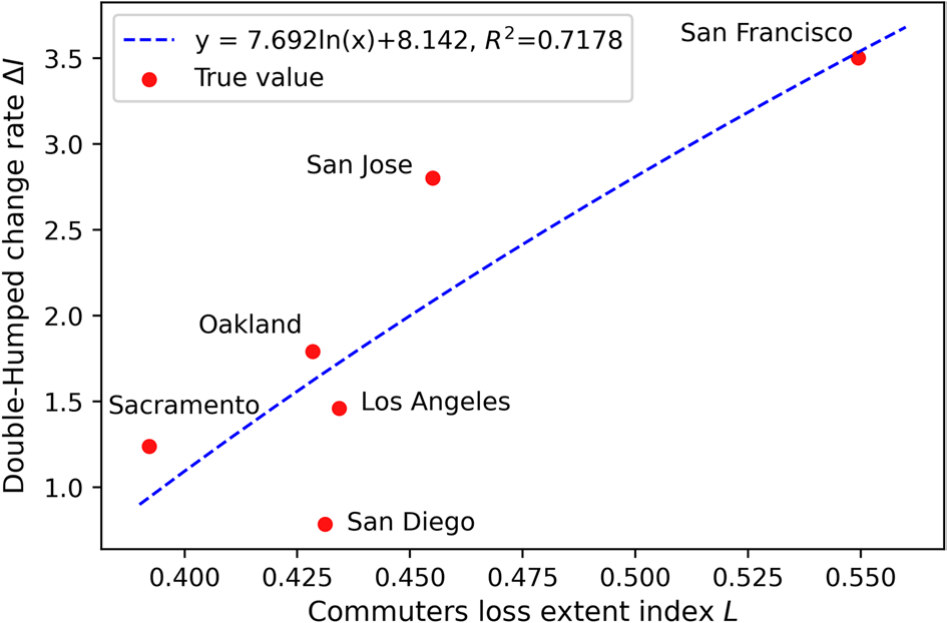
The curve of the relationship between commuters loss extent index *L* and the Double-Humped phenomenon change rate $\Delta I$

According to the Eqs. (7) and (8), the commuters loss extent index *L* and the Double-Humped phenomenon change rate $\Delta I$ for each city are shown in Table 2 and Fig. 7 below.

**Table 2:** The commuters loss extent index *L* and the Double-Humped phenomenon change rate $\Delta I$ for each city

Index	San Diego	San Francisco	Sacramento	Los Angeles	Oakland	San Jose
*L*	0.431	0.549	0.392	0.434	0.428	0.455
$\Delta I$	0.785	3.504	1.238	1.460	1.791	2.803

**Fig. 8:**
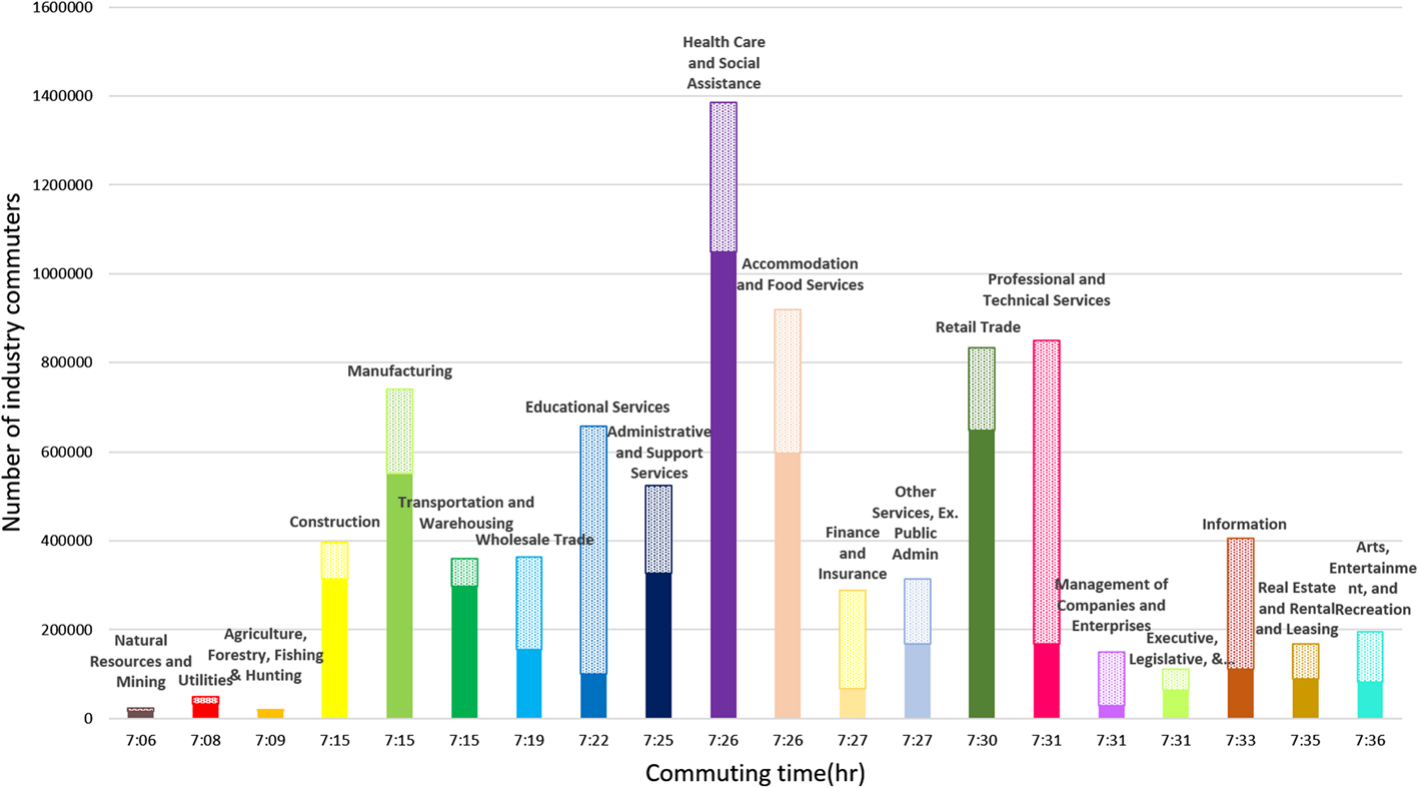
The distribution of morning peak commuters across the state in time and industry before and after the outbreak of COVID-19

From Fig. 7, we observe that city commuters loss extent and Double-Humped phenomenon significance in traffic flow diurnal curves have a positive relationship ($R^2=0.7178$). Unemployment and working from home caused by the pandemic will lead to a sharp drop in the number of commuters during the morning peak period, and the distribution of this decline in time and industry dimensions needs to be further determined. Therefore, we obtain the distribution of commuting departure time of various industries during the morning peak period across the state from the 2010–2012 California Household Travel Survey (CHTS) data, and we assume that there is no significant change in commuting departure time between 2010–2012 and today in various industries. CHTS provides demographic and travel behavior characteristic data for California residents, containing detailed travel behavior information from more than 42,500 households via multiple data-collection methods (National Renewable Energy Laboratory 2017). In addition, through Table 3, we calculate the industry distribution of commuters during the morning peak period across the state before the the outbreak of COVID-19, and commuters loss during the morning peak period in various industries after the outbreak, and the result combined with the distribution of commuting time is shown in Tables 4, 5 and Fig. 8 below, where shaded areas indicate commuter losses across industries.

**Table 3:** Monthly average employment in various industries and corresponding WFHc weight of six California cities before and after the outbreak of COVID-19

Industry	Weight	Year	Los Angeles	Oakland	Sacramento	San Diego	San Francisco	San Jose
Educational services	0.83	2018	377,385	65,110	44,193	107,521	17,267	44,295
		2019	380,928	64,871	45,000	107,108	18,090	44,253
		2020	353,271	59,474	40,300	121,171	15,836	40,283
		2021	354,217	65,375	39,585	94,119	15,376	39,650
Professional and technical services	0.8	2018	291,849	72,688	41,293	139,501	134,522	153,872
		2019	299,007	75,034	43,421	145,935	142,357	162,074
		2020	285,248	72,576	42,848	142,762	142,208	160,359
		2021	286,328	72,934	42,790	145,776	137,136	160,266
Management of companies and enterprises	0.79	2018	59,412	16,806	9675	23,740	23,421	18,989
		2019	62,711	16,677	9965	23,761	20,046	15,279
		2020	59,375	16,066	10,011	23,425	16,841	14,756
		2021	62,075	16,355	10,340	24,056	15,302	14,720
Finance and Insurance	0.76	2018	135,791	17,124	23,947	46,627	42,233	21,320
		2019	134,635	17,486	23,472	46,177	45,584	21,649
		2020	130,736	16,980	23,415	45,694	45,003	22,509
		2021	128,127	16,841	23,125	46,011	44,745	22,671
Information	0.72	2018	203,207	20,209	8760	25,004	46,100	92,336
		2019	210,439	21,468	8586	24,570	51,245	101,110
		2020	187,992	20,332	5270	22,909	53,576	105,668
		2021	206,675	19,566	6991	22,290	53,335	105,470
Wholesale trade	0.52	2018	221,683	37,750	17,622	43,382	15,082	31,445
		2019	218,454	35,897	17,113	43,642	14,219	30,838
		2020	197,648	33,087	16,095	40,817	11,864	28,376
		2021	196,236	32,376	15,562	40,395	9804	27,522
Real estate and rental and leasing	0.42	2018	86,438	10,526	9310	29,158	15,310	15,026
		2019	88,646	10,706	9451	30,092	16,472	15,662
		2020	80,367	9867	9236	28,554	14,644	14,888
		2021	80,498	9551	9040	27,809	14,019	14,417
Executive, legislative and gen government	0.41	2018	43,755	6807	32,730	13,981	6419	7991
		2019	44,285	6806	32,638	14,020	6396	8353
		2020	42,391	6466	32,283	13,595	6282	8580
		2021	40,218	6263	32,147	13,162	6099	7851
Utilities	0.37	2018	27,380	3764	5832	4999	4420	3268
		2019	28,370	3810	5941	4645	4251	3244
		2020	29,051	3916	5984	4725	4331	3299
		2021	27,983	3985	6299	4901	4614	3363
Other services, except public admin.	0.31	2018	155,516	26,408	21,140	52,326	29,488	27,542
		2019	154,961	26,541	21,944	53,514	29,967	27,915
		2020	126,278	21,517	19,346	42,133	23,108	20,995
		2021	119,242	20,554	18,790	39,911	21,726	19,241
Administrative and support services	0.31	2018	257,032	39,183	44,939	82,277	41,499	58,067
		2019	266,552	38,772	42,505	82,372	37,267	58,631
		2020	234,642	34,640	41,554	77,316	31,778	54,314
		2021	236,352	34,008	40,076	81,553	30,097	54,784
Arts, entertainment, and recreation	0.3	2018	104,623	11,448	8692	35,593	14,949	17,694
		2019	107,967	12,840	9181	36,385	15,193	18,128
		2020	72,770	7289	5444	24,444	10,359	11,343
		2021	60,230	5357	4511	21,012	7845	8740
Natural resources and mining	0.25	2018	6619	729	2792	9596	206	3779
		2019	6278	801	2660	10,069	216	3419
		2020	6092	808	2518	9464	227	3256
		2021	6016	855	3163	8601	293	3063
Health care and social assistance	0.25	2018	756,651	114,201	101,752	192,489	75,245	125,422
		2019	777,828	116,705	106,868	199,409	76,855	128,335
		2020	762,685	114,009	106,214	202,820	76,260	128,632
		2021	773,864	116,262	107,809	198,226	78,678	130,947
Manufacturing	0.22	2018	342,206	84,256	20,959	111,597	12,992	167,570
		2019	338,308	84,061	21,391	115,014	13,593	168,650
		2020	313,890	83,522	21,614	113,741	12,197	165,318
		2021	307,305	89,408	21,815	112,706	11,463	164,949
Transportation and warehousing	0.19	2018	219,744	38,101	18,782	36,218	19,759	19,254
		2019	230,039	39,648	20,351	37,705	22,504	19,344
		2020	224,031	41,616	21,188	36,751	20,062	18,960
		2021	229,355	42,862	22,296	39,318	19,297	19,400
Construction	0.19	2018	145,618	49,865	38,327	84,286	23,390	48,930
		2019	149,695	50,684	41,393	84,528	24,045	52,132
		2020	145,978	47,299	42,016	81,689	23,220	49,248
		2021	146,900	47,522	42,438	81,250	21,938	49,628
Retail trade	0.14	2018	424,033	71,451	64,113	149,722	46,496	85,566
		2019	416,640	70,308	63,201	147,564	45,195	83,098
		2020	379,793	61,953	59,102	135,040	38,165	72,310
		2021	390,949	63,134	61,989	137,634	36,525	73,009
Agriculture, forestry, fishing and hunting	0.08	2018	4695	591	2616	9248	193	3581
		2019	4383	679	2487	9713	301	3264
		2020	4410	704	2346	9122	275	3109
		2021	4417	744	2985	8263	285	2914
Accommodation and food services	0.04	2018	441,543	64,547	56,460	176,580	81,651	88,785
		2019	448,709	65,195	58,171	178,949	86,877	89,723
		2020	328,512	45,721	45,413	131,520	49,032	62,349
		2021	294,962	41,408	42,227	117,915	35,678	53,712

The calculation of the morning peak commuting departure time, ranging from 6:00 to 9:00 am, for each industry is the average result for all commuters in that industry, obtained after classifying according to personal work category, travel purpose, working day or not working day, commuting mode and the departure time, range from 5:50 to 8:50 am considering transiting time, where transiting time refers to the time it takes people to travel from home until they use transportation, because in the raw data, the departure time provided is when people leave from home, so we need to account for the 10 min transiting time here. Therefore, the actual commuting departure time of each industry can be regarded as an average distribution centered on the commuting time in Tables 4 and 5, which also means that the actual commuting time distribution is wider, but it does not prevent us from judging the commuting time sequence of each industry according to the result in tables.

**Table 4:** The distribution of morning peak commuters across the state in time and industry before outbreak

Industry	Commuting time (*hr*)	Number of commuters
Natural resources and mining	7:06	23,582
Utilities	7:08	49,962
Agriculture, forestry, fishing and hunting	7:09	20,876
Construction	7:15	396,447
Manufacturing	7:15	740,299
Transportation and warehousing	7:15	360,725
Wholesale trade	7:19	363,564
Educational services	7:22	658,011
Administrative and support services	7:25	524,548
Health care and social assistance	7:26	1,385,880
Accommodation and food services	7:26	918,595
Finance and insurance	7:27	288,023
Other services, ex. public admin	7:27	313,631
Retail trade	7:30	833,694
Professional and technical services	7:31	850,777
Management of companies and enterprises	7:31	150,241
Executive, legislative and gen government	7:31	112,091
Information	7:33	406,517
Real estate and rental and leasing	7:35	168,399
Arts, entertainment, and recreation	7:36	196,347

**Table 5:** The distribution of morning peak commuters loss across the state in time and industry after outbreak

Industry	Commuting time (*hr*)	Commuters loss
Natural resources and mining	7:06	7132
Utilities	7:08	17,643
Agriculture, forestry, fishing and hunting	7:09	2729
Construction	7:15	81,212
Manufacturing	7:15	187,560
Transportation and warehousing	7:15	62,841
Wholesale trade	7:19	208,460
Educational services	7:22	558,115
Administrative and support services	7:25	196,541
Health care and social assistance	7:26	337,376
Accommodation and food services	7:26	320,560
Finance and insurance	7:27	220,183
Other services, ex. public admin	7:27	145,709
Retail trade	7:30	185,259
Professional and technical services	7:31	681,717
Management of companies and enterprises	7:31	120,376
Executive, legislative and Gen government	7:31	48,742
Information	7:33	294,591
Real estate and rental and leasing	7:35	78,140
Arts, entertainment, and recreation	7:36	114,882

From Fig. 8, we notice that Health Care and Social Assistance, Accommodation and Food Services, Professional and Technical Services, Retail trade, manufacturing, Educational Services, and Administrative and Support Services account for the top seven of the pre-pandemic morning peak period commuting industries, and the commuting time distribution of these industries is located in the middle of the morning peak period, and this period is also mixed with other industries that account for a relatively small proportion, such as Transportation and Warehousing and Wholesale Trade, which explains why the Double-Humped phenomenon occurs in the morning peak period traffic flow diurnal curves.

In addition, we find that Professional and Technical Services, Educational Services, Health Care and Social Assistance, Accommodation and Food Services, and Information account for the top five industries that experienced the most commute losses during the morning peak period after the outbreak of the pandemic, and their commuting time distributes in the middle of the morning peak period. The commuting time of the seven industries with the least proportions, such as Natural Resources and Mining, Utilities, Agriculture, Forestry, Fishing and Hunting, Construction, Executive, Legislative and General Government, Real Estate, Rental and Leasing and Arts, Entertainment and Recreation, all distributes at the front and back of the morning peak period. While this is a temporal phenomenon, it is also spatial, as those sectors in addition to being less demanding of mid-peak travel, also have less centralised workplaces. We don’t expect natural resource extraction, energy production and water treatment, farming, construction, or most arts (aside perhaps from live performances) in particular to be downtown oriented.

This distribution pattern of commuters loss makes the traffic flow in the middle part of the traffic flow diurnal curve drop more severely than on both sides, and thus the acuteness of the peaks and valley of the traffic flow diurnal curve become more significant. In addition, more traffic drop between the two peaks will make the two peaks more prominent. The Double-Humped phenomenon in traffic flow diurnal curves became more pronounced after the outbreak of COVID-19, and different cities have different changes in the Double-Humped phenomenon due to the extent of commuter loss by industry sector.

## Conclusions

In this paper, we observe a bifurcation phenomenon in the traffic flow pattern that was formerly mostly a single-peaked pattern during the morning peak commute period in the freeway network after the outbreak of COVID-19. We extend the previous study (Gao and Levinson 2021) of the Double-Humped phenomenon in traffic flow diurnal curve to six cities in California. Through the collection and analysis of traffic flow and speed data of the freeway network in these cities, we corroborated the results from the freeway network in the Minneapolis-St. Paul region (Gao and Levinson 2021), and provided evidence. We observe the Double-Humped phenomenon in the traffic flow diurnal curve became more significant after the COVID-19 outbreak, and saw the decrease in unit travel time after the outbreak, which means that the congestion in the morning peak was alleviated due to the pandemic.

The reason why the Double-Humped phenomenon has become more obvious is due to the change in the composition of morning peak commuting. The significance of the Double-Humped phenomenon in different cities after the outbreak is also different, which is related to the degree of commuting losses in the city ($R^2=0.7178$). In order to better explain this phenomenon, we collected monthly average employment data and California Household Travel Survey (CHTS) data, and analyzed the data to obtain the distribution of commuting time during the morning peak of various industries across the state and corresponding commuters loss. From the results, we found that the industries that suffered the most commuters loss (i.e. had the most work from home and unemployment) from the pandemic traveled in the middle of the morning peak (6:00–9:00 am), which we believe is an important causal factor for this remarkable emergence of the Double-Humped peak.
